# Supplementary material for: Efficient Hydrolysis of Earthworm Protein and the Lipid-Lowering Mechanism of Peptides in the Hydrolysate
Source: Foods. 2025 Jul 1;14(13):2338. doi: 10.3390/foods14132338 (PMC12249373; doi:10.3390/foods14132338)
Supplement: Supplementary file 1 [file foods-14-02338-s001.zip › foods-3719225-supplementary.pdf]

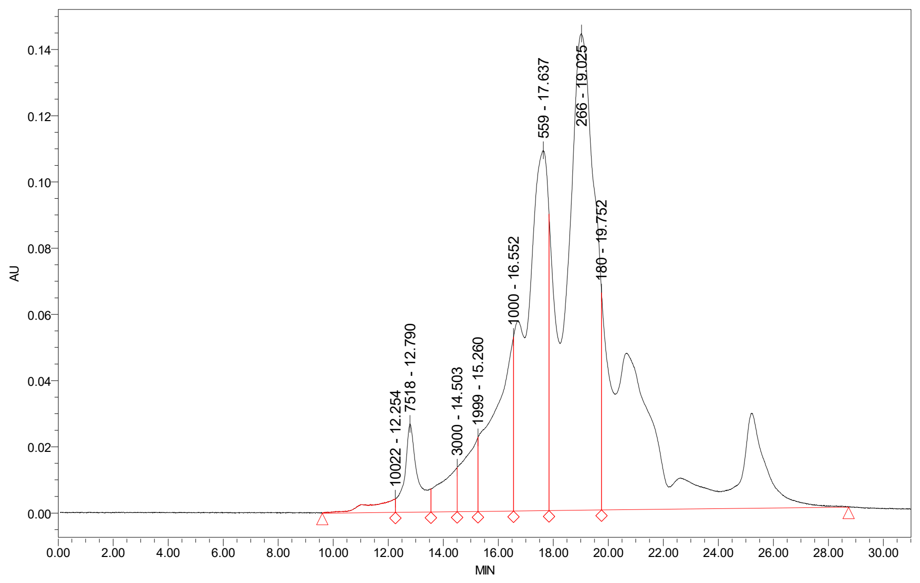

Fig. S1 Volume exclusion high performance liquid chromatography of EPH.

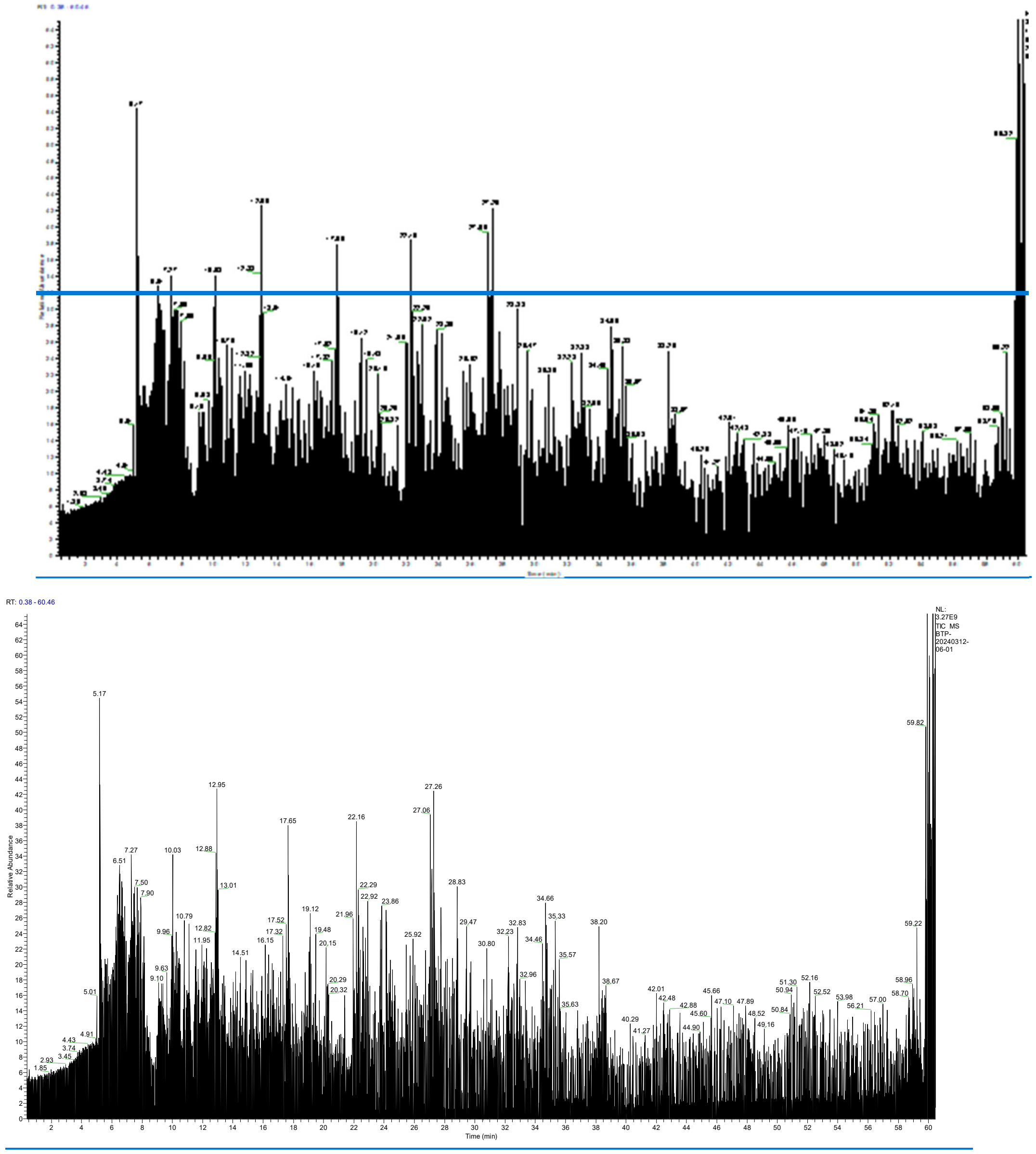

Fig.S2 Total ion flow chromatogram of EWP

Quality control results

The quality of RNA is of pivotal significance for the execution of RNA-Seq experiments. As shown in Table S1, the findings demonstrated that the detection limit of the sequencer was attained when the total RNA concentration of the samples in each group was ≥ 101.0 ng/μL. The results of the Agilent 2100 integrity assay were as follows: The baseline was flat, the peak pattern was normal, and the RNA integrity value (RIN) was ≥ 8.70, which met the sequencing requirements. Subsequently, the mRNA was screened, fragmented and reverse transcribed in order to generate an RNA-Seq cDNA library. The raw sequence data of each sample is transformed into valid data that can be used for comparative analyses after the filtering process (Table S2). The base quality score values of each group are situated in the green high-quality area, indicating that the reliability of the sequencing data results (processed by Fast QC software) is high. Subsequently, nine samples were analyzed for sequence comparison, and the percentage of sequenced sequence locus of each sample reached more than 80%, with high quality data analysis and reliable results (Table S3). Concurrently, the expression levels of all genes were quantified employing String Tie software, with FPKM values ranging from 0.1 to 3.75, 3.75 to 15, and FPKM values > 15 representing low, medium, and high abundance, respectively (Table S4).

Table S1 Results of RNA-Seq cDNA library concentration and RIN values

Library Name	Concentration(ng/μL)	Volumen(μL)	RIN
HFT-1	164.0	80.0	9.20
HFT-2	136.0	80.0	9.10
HFT-3	133.0	80.0	9.00
EWP-1	163.0	80.0	9.30
EWP-2	101.0	80.0	9.20
EWP-3	153.0	80.0	9.20

Table S2 The yield statistics of the clean reads data

Sample	Raw reads	Clean eads	Q20 rate (%)	GC (%)
HFT-1	48,708,914	48,636,602	98.69	49.09
HFT-2	41,977,838	41,915,220	98.47	48.75
HFT-3	53,707,058	53,627,826	98.43	48.65
EWP-1	41,331,090	41,273,932	98.47	48.92
EWP-2	59,797,854	59,708,016	98.71	48.91
EWP-3	42,004,846	41,947,750	98.41	48.33

Table S3 Results of the sequence alignment analysis

Sample	Total reads	Total mapped	Multiple mapped	Uniquely mapped
HFT-1	48636602	47618939 (97.91%)	3943197 (8.11%)	43675742 (89.80%)
HFT-2	41915220	40915240 (97.61%)	3294903 (7.86%)	37620337 (89.75%)
HFT-3	53627826	52375868 (97.67%)	4233544 (7.89%)	48142324 (89.77%)
EWP-1	41273932	40283647 (97.60%)	3301436 (8.00%)	36982211 (89.60%)
EWP-2	59708016	58393292 (97.80%)	4817290 (8.07%)	53576002 (89.73%)
EWP-3	41947750	40900230 (97.50%)	3276744 (7.81%)	37623486 (89.69%)

Table S4 Statistics of gene expression level

Sample	0-0.1	Ratio (%)	0.1-3.75	Ratio (%)	3.75-15	Ratio (%)	> 15	Ratio (%)
EWP-1	23821	55.76	9128	21.37	5689	13.32%	4081	9.55
EWP-2	23918	55.99	9198	21.53	5668	13.27%	3935	9.21
EWP-3	23824	55.77	9093	21.29	5682	13.30%	4120	9.64
HFT-1	23669	55.41	9293	21.75	5700	13.34%	4057	9.50
HFT-2	23673	55.42	9212	21.56	5722	13.39%	4112	9.63
HFT-3	23668	55.40	9220	21.58	5703	13.35%	4128	9.66

Table S5 Statistics of the number of differential genes

Pairs	Up	Down
HFT_vs_EWP	214	304

Table S6 Primers designed for qPCR

Primers	Sequence (5' to 3')
IL6-F	CCTGAACCTTCCAAAGATGG
IL6-R	TTCACCAGGCAAGTCTCCTC
CXCL8-F	ACTGAGAGTGATTGAGAGTGGAC
CXCL8-R	AACCCTCTGCACCCAGTTTTC
JUN-F	CGGAGACAGCCACCAACTC-
JUN-R	GTTGGCGACTCCTGTTCTGA
SPP1-F	CAGTGATTTGCTTTTGCCTGT-
SPP1-R	GGTGATGTCCTCGTCTGTA
CCR1-F	GCTGTGGTGGTCATTGTCAT
CCR1-R	TGGTGTCCTGGAGTTGAGTG
IL1A-F	CCTGAAGCCCTTGTTGTAGG
IL1A-R	GGCAGACTCAAATTCCAGCA
CXCL2-F	GCCAGTGCTTCTGTGCAGTA
CXCL2-R	TGGTTCTTCCGTTGAGGGA
LCN2-F	TGTGCTGGACTACAACCAGTTC
LCN2-R	GGCACACTGAACGGTACTGT
LOX-F	CACCAACGAGTTCAAGGTGG
LOX-R	CCAGGGTGTCCAGGTAGATG
MMP1-F	GAAATGGGCTTTGAAGGTGA
MMP1-R	CAGGGTTGATCCAGAACTCC
GAPDH-F	TTTGTCAAGCTCATTTCCTGGTATG
GAPDH-R	TGGGATAGGGCCTCTCTTGC
